# Decoding the Duality of Antinutrients: Assessing the Impact of Protein Extraction Methods on Plant-Based Protein Sources

**DOI:** 10.1021/acs.jafc.4c00380

**Published:** 2024-05-23

**Authors:** Maria Lilibeth Manzanilla-Valdez, Zidan Ma, Martin Mondor, Alan Javier Hernández-Álvarez

**Affiliations:** † Food Science and Nutrition, University of Leeds, Leeds, LS2 9JT, United Kingdom; ‡ Saint-Hyacinthe Research and Development Centre, Agriculture and Agri-Food Canada, Saint-Hyacinthe, Quebec Canada, J2S 8E3; § Institute of Nutrition and Functional Foods (INAF), Université Laval, Quebec, Quebec G1V 0A6, Canada

**Keywords:** antinutritional factors, protein extraction, plant proteins, wet extraction, protein digestibility, dry fractionation

## Abstract

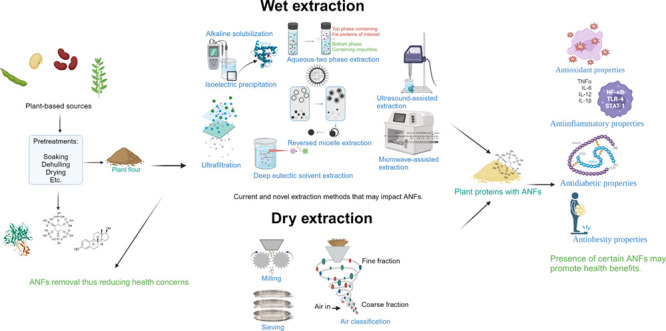

This review aims to provide an updated overview of the effects of protein extraction/recovery on antinutritional factors (ANFs) in plant protein ingredients, such as protein-rich fractions, protein concentrates, and isolates. ANFs mainly include lectins, trypsin inhibitors, phytic acid, phenolic compounds, oxalates, saponins, tannins, and cyanogenic glycosides. The current technologies used to recover proteins (e.g., wet extraction, dry fractionation) and novel technologies (e.g., membrane processing) are included in this review. The mechanisms involved during protein extraction/recovery that may enhance or decrease the ANF content in plant protein ingredients are discussed. However, studies on the effects of protein extraction/recovery on specific ANFs are still scarce, especially for novel technologies such as ultrasound- and microwave-assisted extraction and membrane processing. Although the negative effects of ANFs on protein digestibility and the overall absorption of plant proteins and other nutrients are a health concern, it is also important to highlight the potential positive effects of ANFs. This is particularly relevant given the rise of novel protein ingredients in the market and the potential presence or absence of these factors and their effects on consumers’ health.

## 1 Introduction

Over the past decades, plant-based foods have gained stronger scientific support for their potential health benefits. These include a decrease in the risk of chronic noncommunicable diseases, such as cancer, type 2 diabetes, hypertension, and dyslipidaemia.^1^

In plant materials like legumes, nuts, and cereals, naturally occurring macronutrients include proteins, carbohydrates, fibers, minerals, and vitamins, which provide nutritional benefits. Naturally occurring antinutritional factors (ANFs) such as cyanogenic glycosides, lectins, saponins, tannins, phytic acid, trypsin inhibitors, and oxalates are also found in plant materials. ANFs are known to have some adverse effects on human health but they may also provide health benefits. Common health concerns associated with ANFs include vomiting, bloating, and reduced bioavailability of minerals and proteins. However, the health benefits of ANFs are also significant, including scavenging free radicals, prevention of type 2 diabetes, anti-inflammatory properties, and anticancer attributes.^2,3^

Among the various macronutrients derived from plant sources, plant-based proteins are increasingly gaining prominence. The advantages of plant proteins, particularly as potential alternative sources of protein, lie primarily in their ability to meet the future protein demands of a growing global population, as well as in offering substantial environmental benefits. Compared to animal protein sources, plant protein sources emit less greenhouse gas emissions and require less land.^4^ Extraction/purification technologies used to recover proteins from plant sources can be classified in two main categories: (1) conventional methods such as alkaline extraction, isoelectric precipitation, dry fractionation, solvent extraction (organic or inorganic), and salt extraction (salt in and out), and (2) novel methods such as membrane processing, enzyme-assisted extraction, reverse micelle, microwave-assisted extraction, ultrasound-assisted extraction, subcritical water extraction, high pressure assisted-extraction, pulse electric field assisted extraction, and deep eutectic solvent.^5^ Despite numerous research studies conducted over the past 30 to 40 years exploring novel methods like membrane technologies, their application at an industrial scale is only just beginning.^5,6^

Currently, there is a notable gap in information concerning the presence or absence of ANFs in plant-based protein isolates, concentrates, and protein-rich fractions. Moreover, there has been no comprehensive review to elucidate the effects of various protein extraction/recovery procedures employed in the production of these ingredients, particularly in terms of their influence on either reducing or increasing ANFs. Therefore, the purpose of this review is to offer a detailed overview of the existing evidence regarding the presence of ANFs in plant protein ingredients. This review will also critically analyze and try to arrive at an understanding of the impact of different protein extraction methodologies on ANF content, as well as explore the underlying mechanisms of protein extraction and fractionation.

## 2 Protein Extraction and ANFs

Currently, the demand for plant proteins represents one of the most rapidly growing segments of the food industry. This surge is driven by several factors: (1) environmental concerns associated with animal-based proteins and issues surrounding animal welfare, (2) health benefits from plant protein foods and dietary patterns, and (3) the need to feed a growing global population.^7^ Consequently, there has been increased focus on healthier, plant-based protein ingredients. Current research primarily targets aspects that evaluate protein quality and protein content such as bioavailability and digestibility in protein ingredients.^8^

However, ANFs in protein concentrates/isolates are often overlooked. Studies have shown that ANFs, such as phytic acid and trypsin inhibitors, are present in higher concentrations in protein concentrates/isolates than in raw plant flours. The levels of ANFs also depend on the methods used for protein extraction/recovery. Generally, protein extraction methods are classified as wet or dry. The traditional and most studied wet extraction method is alkaline solubilization, followed by isoelectric precipitation. In this method, proteins are solubilized in an aqueous solution under alkaline conditions and then precipitated by adjusting the pH to their isoelectric point.^7^ Alkaline solubilization has also been combined with membrane technologies like ultrafiltration for protein recovery.^9,10^ Innovative wet extraction methods, such as two-phase extraction (including aqueous two-phase extraction and reversed micelle extraction), rely on the incompatibility of two aqueous phases and differential protein solubility. Other novel wet extraction techniques employ energies such as ultrasound, microwaves and the use of biomolecules such as enzymes to enhance protein extraction.^7^ These techniques can disrupt plant cell structures, facilitating the penetration of extraction solvents into cells for more effective extraction.

In addition to alkaline solubilization/isoelectric precipitation, dry extraction technology is the most common method used to extract proteins from plants. Dry extraction technologies separate protein-rich fractions based on particle size, using milling and airstream processes to mechanically isolate proteins from other cellular components like starch.^11^

Due to the varied mechanisms of these extraction technologies, the content and activity levels of ANFs are affected differently. In addition, there is a lack of research regarding the effect of these diverse extraction technologies used to formulate protein ingredients with specific compounds, and their potential to impact human health.

## 3 Classification of Antinutritional Factors and Potential Effects on Health

**Figure 1:**
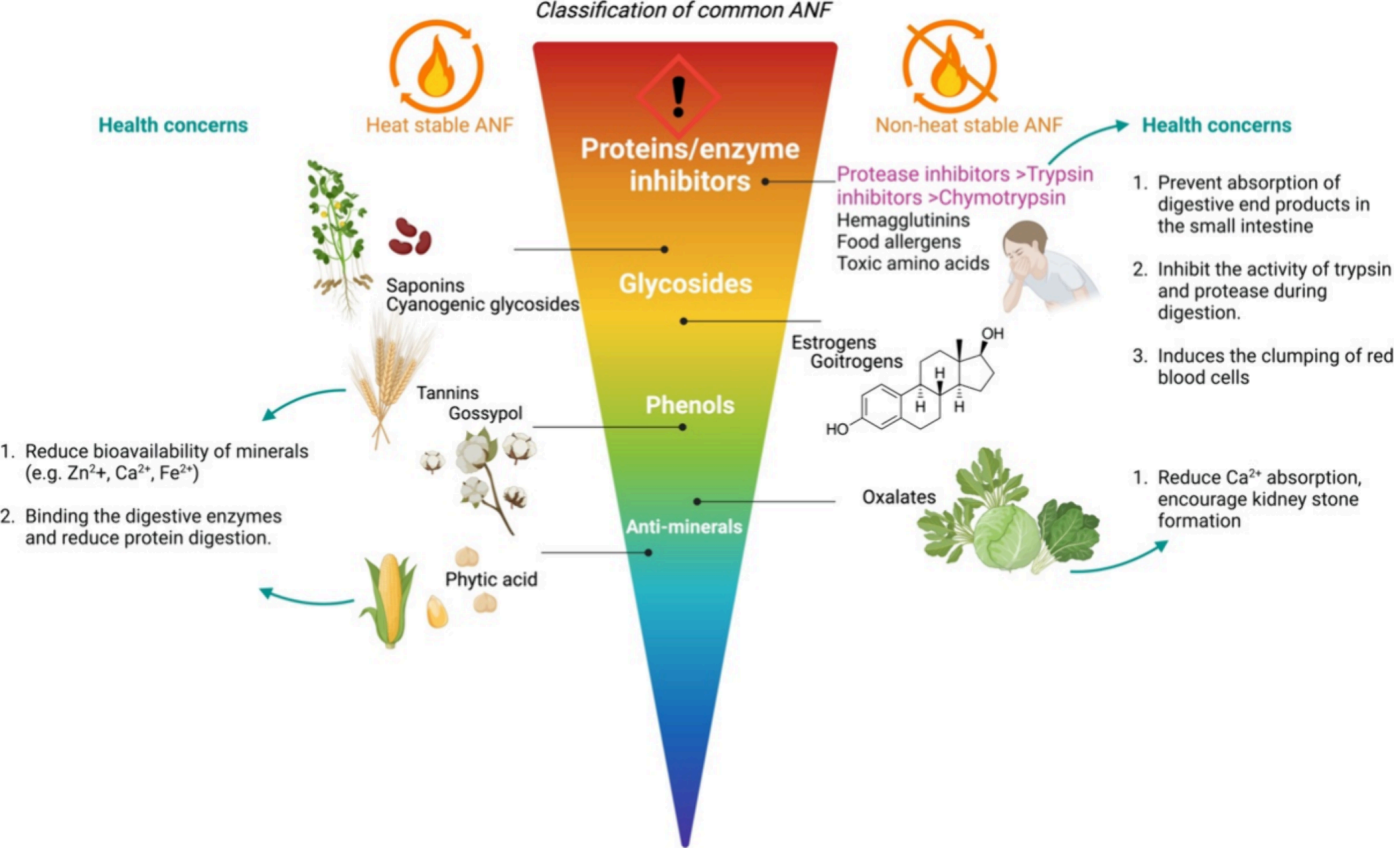
Classification of common ANFs and their corresponding heat stability.

The methods for classifying ANFs vary. According to Gemede and Ratta,^12^ ANFs can be divided into a heat resistant group and a nonheat resistant group. The heat stable group includes “phytic acid, tannins, alkaloids, saponins,” while the common ANFs found in the nonheat stable groups are lectins, protease inhibitors, and oxalates. Another classification method is based on ANFs’ chemical structures. Liener^13^ classified ANFs into protein/enzyme inhibitors, glycosides, phenols, and other factors that reduce the bioavailability of minerals (e.g., phytic acid, oxalates).^12,13^ Well-studied ANFs are classified and listed in Figure 1 where their health concerns are represented.

On the basis of the classification of ANFs, different ANFs have different adverse effects on human and animal health (Table 1).^14^ The ANFs in the protein/enzyme inhibitor group generally have negative effects on food digestion. For example, α-amylase inhibitors slow starch digestion, and trypsin inhibitors and protease inhibitors inhibit the activity of trypsin and proteases during protein digestion. A specific class of component in the ANF protein/enzyme inhibitor group are lectins, which have a similar digestion-inhibiting property to that of other enzyme inhibitors. However, this property of lectins does not directly inhibit the digestive enzymes. Instead, lectins interfere with nutrient breakdown and absorption by reversibly binding to sugars and/or glycoproteins on gut wall surface cells.^15^ Lectins specifically recognize and bind sugar moieties present on the surface of erythrocytes which leads to cross-linking of the cells and a formation of cell clumps called agglutinates, also known as red blood cell agglutination.^15^ Animal models have demonstrated that high lectin doses can interact with intestinal epithelial cells and lead to an increase in intestinal epithelial permeability and interfere with nutrient absorption.^16^

**Table 1:** Description, Side Effects, and Biological Positive Outcomes of ANFs

**ANFs**	**description**	**side effects**	**biological positive outcomes**	**references**
**Anthocyanins**	Flavonoids with two aromatic rings and heterocyclic ring	• Impairs protein absorption	• Antioxidant capacity	(85,86)
			• Cardiovascular protection	
			• Anti-inflammatory activity	
**Cyanogenic glucosides**	Cyanogens are glycosides of glucose with a cyanide aglycone	• Inactivates cytochrome oxidase in the mitochondria and binds to Fe^2+^	• Antibacterial effect	(14)
		• Decrease in oxygen to organ tissue		
**Lectins**	Oligomeric polypeptide with three subunits α, β, and γ	• Hemagglutination	• Antitumor effect	(74,87)
		• Stimulates antibody production of T-cells	• Antimicrobial, antifungal, antiviral and antibacterial	
**Oxalates**	Water-soluble salt formed from oxalic acid	• Inhibits the metabolism of Ca^2+^ and Mg^2+^	• not available	(14,69)
		• Formation of kidney stones		
		• Could act as blood clotting factor		
**Phenolics**	Produced from phenyl propanoid or shikimate pathway	• Decrease in bioavailability of amino acids	• Inhibit lipid oxidation	(88,89)
			• Antioxidant capacity	
**Phytic acid**	Major storage form of phosphorus	• Chelation of minerals (Mg^2+^, Ca^2+^, Fe^2+^, and Zn^2+^)	• Cardiovascular protection effect	(74,87)
		• Chelation of proteins and reduced bioavailability	• Prevention of kidney stone formation	
			• Decreased risk of colon cancer	
**Saponins**	Triterpenoids or steroidal glycosides	• Lysis of red blood cells	• Triterpenoids can act as antioxidant agents	(74)
		• Decreases protein, mineral and vitamin absorption	• Reduced total cholesterol	
		• Can lead to hypoglycemia	• Antimicrobial properties	
		• Severe diarrhea		
**Tannins**	Water-soluble phenolic compounds, three main categories: hydrolyzable, condensed, and complex	• Inhibition of hydrolytic enzymes	• Moderate amounts act as antioxidant agents	(74)
		• Reduces protein digestibility		
**Trypsin inhibitors**	Proteins	• Inhibit protein digestion and amino acid absorption	• Prevents pancreatic acute disease	(74,87)
			• Pancreatic hyperplasia	

ANFs interacting with minerals include phytic acid, phenolic compounds, and oxalates, which reduce the absorption of minerals, including iron and calcium. Phytic acid can form complexes with minerals such as copper, zinc, manganese, iron, and calcium. These complexes are insoluble and cannot be hydrolyzed by human digestion enzymes, therefore hindering mineral absorption.^17,18^ The presence of phytic acid in protein ingredients may potentially promote health benefits. Several studies have reported the beneficial health functions of phytic acid in the human body including antioxidant activity, diabetes prevention, anti-inflammatory properties, and colon cancer regulation.^3^ However, there is a lack of research regarding the potential benefits of residual phytic acid in extracted plant protein ingredients. Consequently, a comparative analysis of the health benefits between these extracted plant proteins and whole plant materials could be conducted.

Similar to phytates, soluble oxalates bind to minerals, thus hindering mineral absorption. Soluble oxalates can also be released at the gastrointestinal pH, forming insoluble salts, which can cause kidney stone and renal failure.^19^

Similarly, phenolic compounds may also interfere with mineral absorption.^14^ For example, tannins have the capacity to form covalent binding and hydrogen binding with protein (causing protein precipitation), vitamins, and minerals.^17^ However, the antinutritional effects (e.g., reduced mineral absorption) of phenolic compounds is also dependent on diet and the amount of food consumed.^18^ Thus, some phenolic compounds such as chlorophenols, nonyphenols and BPAs (bisphenol A) are known for being genotoxic and hormonal disrupting agents.^20^ These compounds can cause cancer by blocking hormonal function and can generate phenoxy radicals, which inhibit the synthesis of ATP cells.^20^

Furthermore, during digestion, tannins can significantly influence the pH mechanism. For instance, under highly acidic conditions (pH 1.0–3.0) during the gastric phase, tannic acid maintains its ability to bind proteins. However, at a higher pH (>6.5), typical of the intestinal phase, there is an enhancement in the hydrolysis of tannic acid, which concurrently reduces its protein-binding capacity.^21^

## 4 Mechanisms of Action of ANFs and Effects of Protein Extraction on ANFs Levels

### 4.1 Trypsin Inhibitors

**Figure 2:**
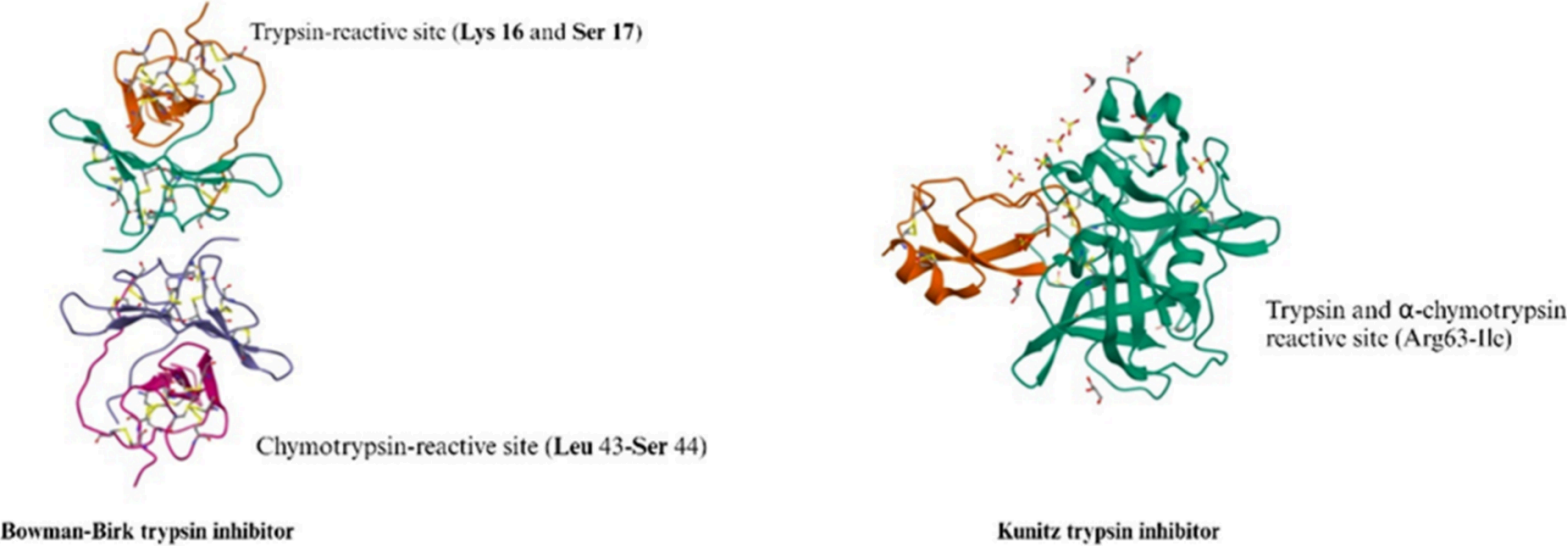
Bowman-Birk trypsin inhibitor and Kunitz trypsin inhibitor, and their corresponding reactive sites.^26,27^

Kunitz trypsin and Bowman-Birk inhibitors are two well-studied trypsin inhibitors in plants. Kunitz trypsin inhibitor acts as an endogenous proteinase regulator in plants. It is a stable globulin type protein (21.5 kDa) with 181 amino acid residues.^22^ The enzymatic inhibition property of Kunitz trypsin inhibitor mainly stems from its amino acid composition and its structure. The primary structure consists of 181 amino acid residues and 2 disulfide bridges. The secondary structure has a spherical shape, stabilized by the hydrophobic side chains, and 12 antiparallel β-strands.^23^ The Kunitz trypsin inhibitor has one reactive site at Arg 63-Ile (Figure 2). From its primary structure, cleaving of the two disulfide bonds between Cys39-Cys86 and Cys138-Cys145 is the key to inactive Kunitz trypsin inhibitor.^23^ The Kunitz soybean trypsin inhibitor is regarded as a specific allergen. Food allergens are normally resistant to the acidic gastric environment and pepsin proteolysis; thus, their stability helps to maintain allergic epitopes and allergenic potential.^24^ Similarly, Roychaudhuri et al.^25^ simulated acidic gastric digestion and found that pepsin proteolysis and acidic pH cannot totally denature the Kunitz soybean trypsin inhibitor. In the acidic environment, the Kunitz soybean trypsin inhibitor presents an acid-induced molten state. However, the acid-induced molten state was still able to cross the gastrointestinal membrane barrier and trigger IgE response.^25^

Bowman-Birk trypsin inhibitor consists of 71 amino acids and 7 disulfide bonds. They also have two reactive sites (Lys16-Ser17 and Leu43-Ser44) that bind trypsin and chymotrypsin (Figure 2).^26^

Protein extraction affects trypsin inhibition activity (TIA) differently. Studies related to protein extraction and its effects on trypsin inhibitors is summarized in Table 2. Wet extraction seems to decrease TIA, while dry extraction results in an increase in TIA. The increase in TIA observed for dry extraction processes (such as air classification, milling coupled to air classification) results from the aggregation of trypsin inhibitor along with the fractionation/protein concentration process, which limits their denaturation.^28^ Several wet extraction/purification methods such as alkaline extraction, isoelectric precipitation, and membrane processing (ultrafiltration) are known to have positive effects on reducing TIA. Barbana and Boye^28^ reported that TIA in lentil protein isolates/concentrates was lower than in raw flour, and red lentil protein precipitation at pH 4.3 reduced TIA from 0.94 TIA mg^–1^ flour to 0.17 TIA mg^–1^ protein concentrate.^29,30^ Similarly, the TIA of green lentil was also reduced by protein precipitation (pH 4.3) from 1.94 TIA mg^–1^ flour to 0.66 TIA mg^–1^ protein concentrate.^29^ Regarding the isoelectric precipitation method, as trypsin inhibitors are water-soluble proteins, these are first solubilized in the extraction medium (water), and due to the precipitation pH normally chosen at the protein’s isoelectric point (∼pH 4.5), both the Bowman-Birk and Kunitz trypsin inhibitors can be precipitated, as the isoelectric point of the Bowman-Birk trypsin inhibitor is at pH 4.0, while the one for the Kunitz trypsin inhibitor is at pH 4.5.^22,31^

**Table 2:**
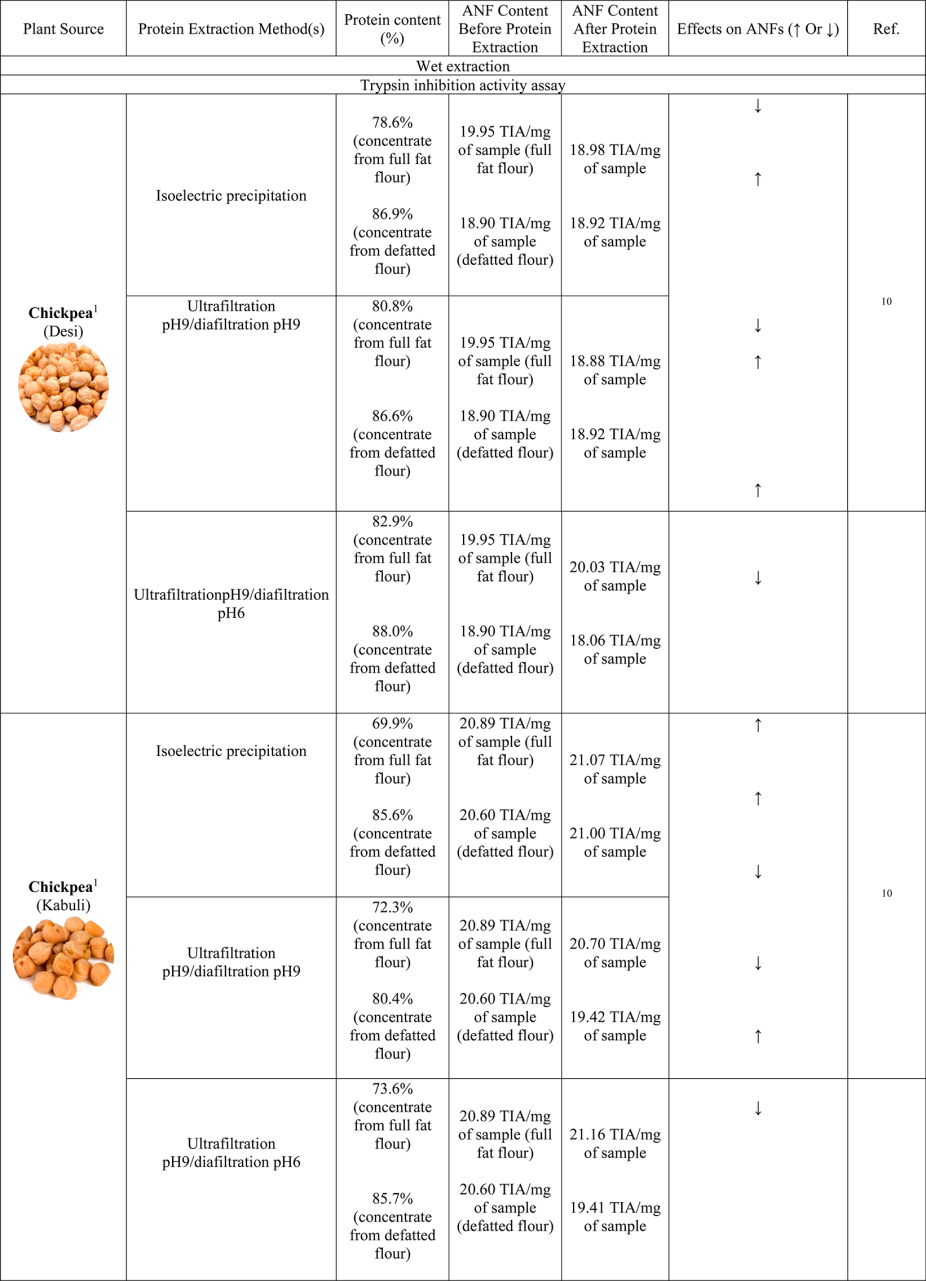

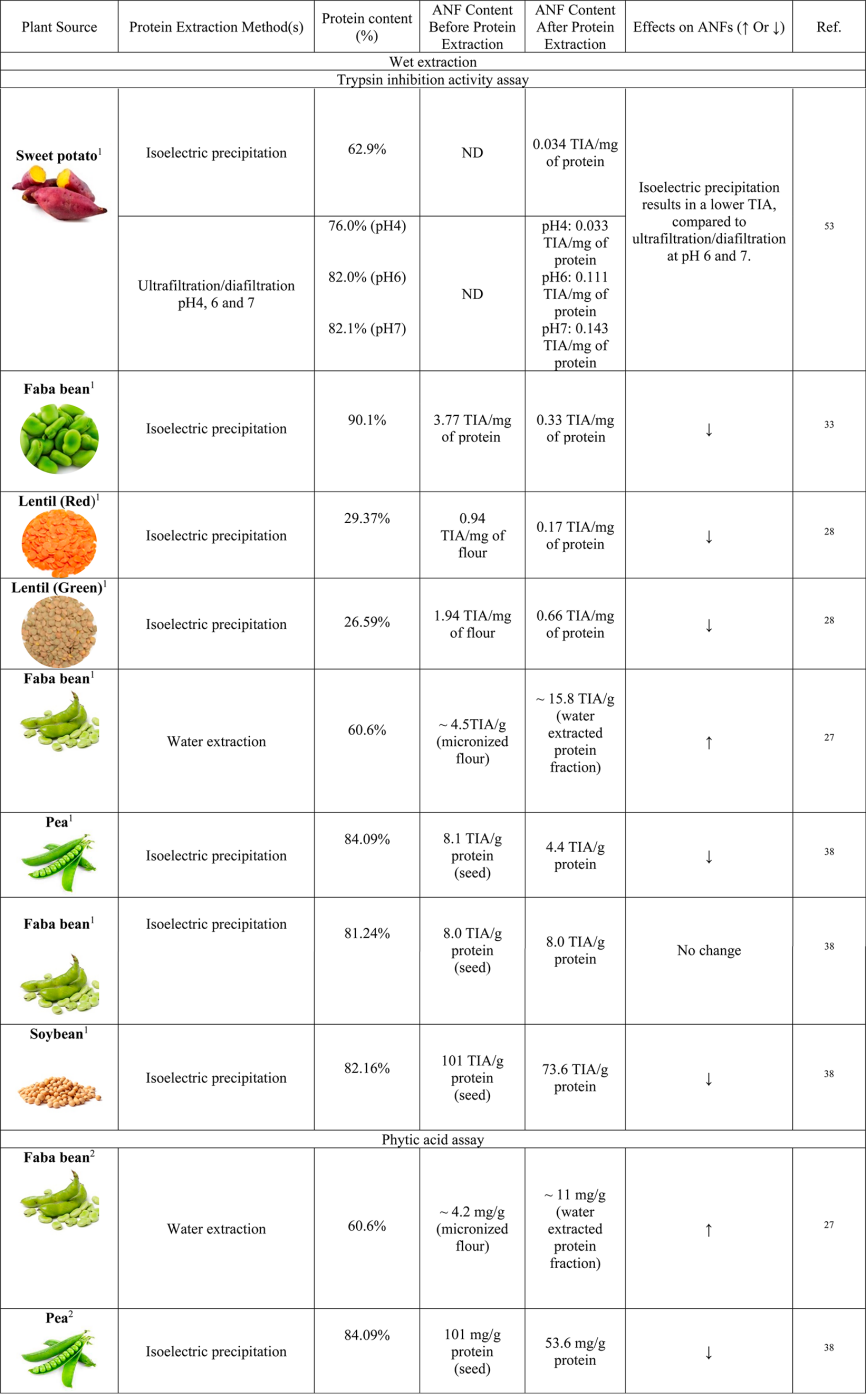

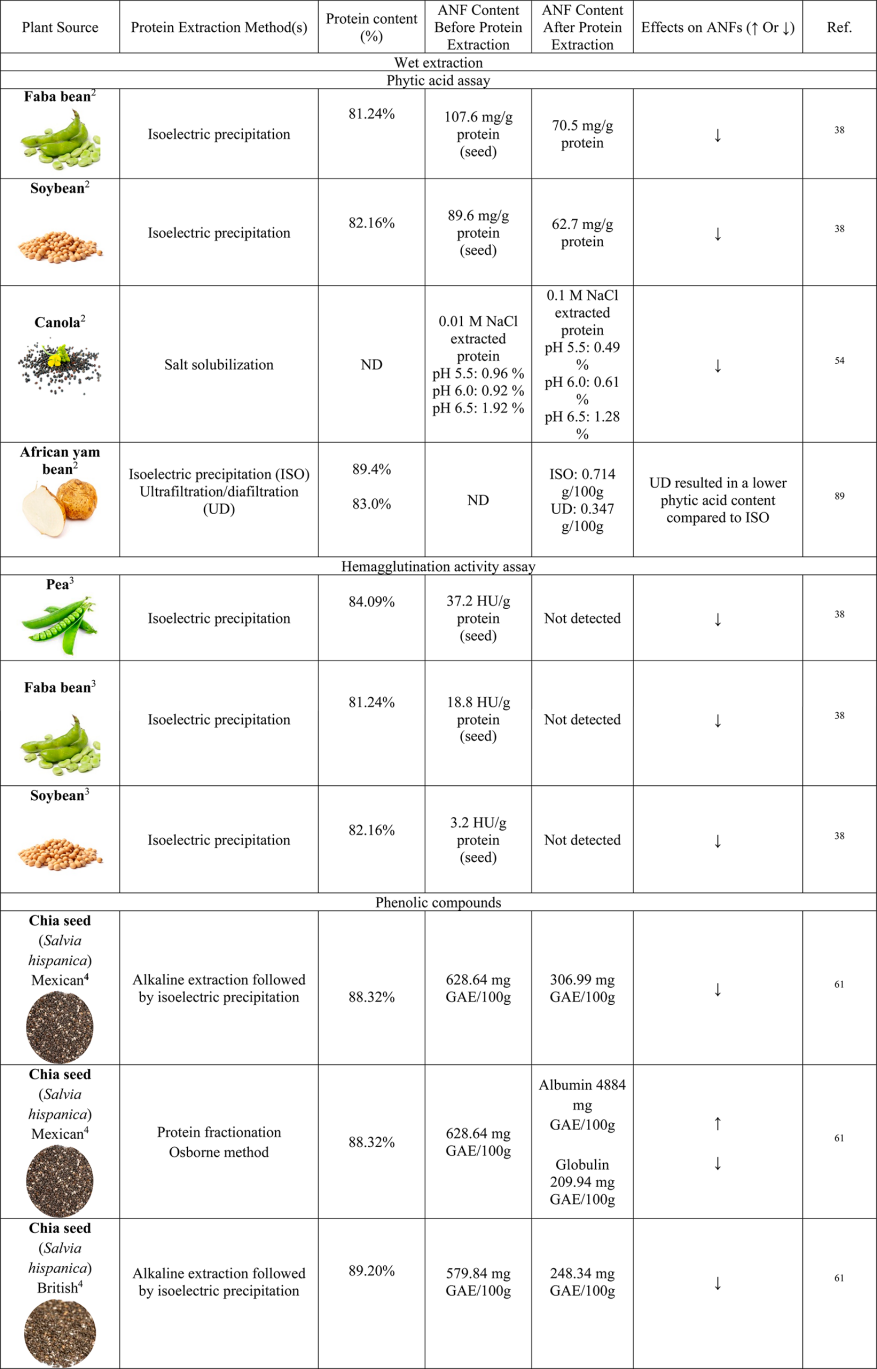

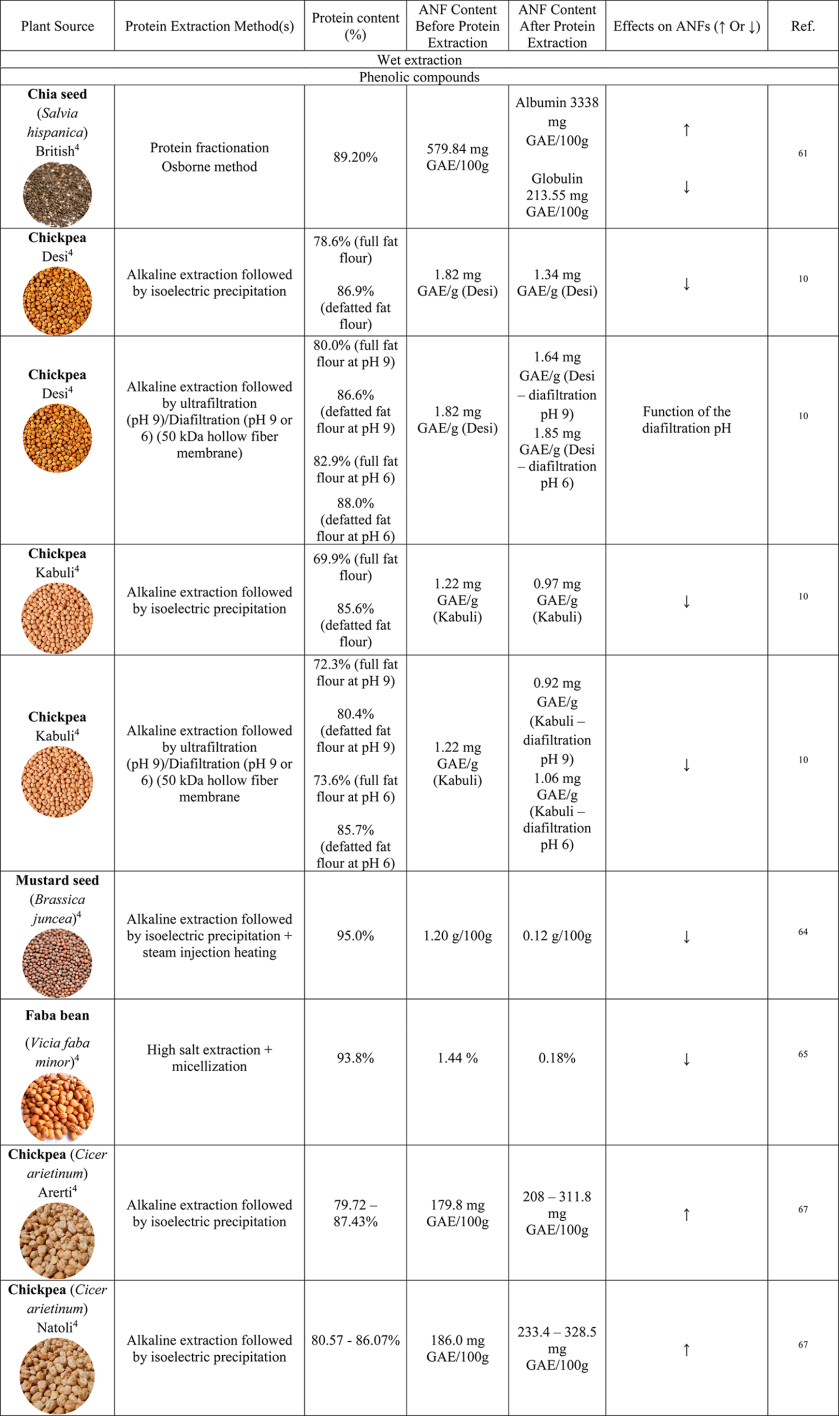

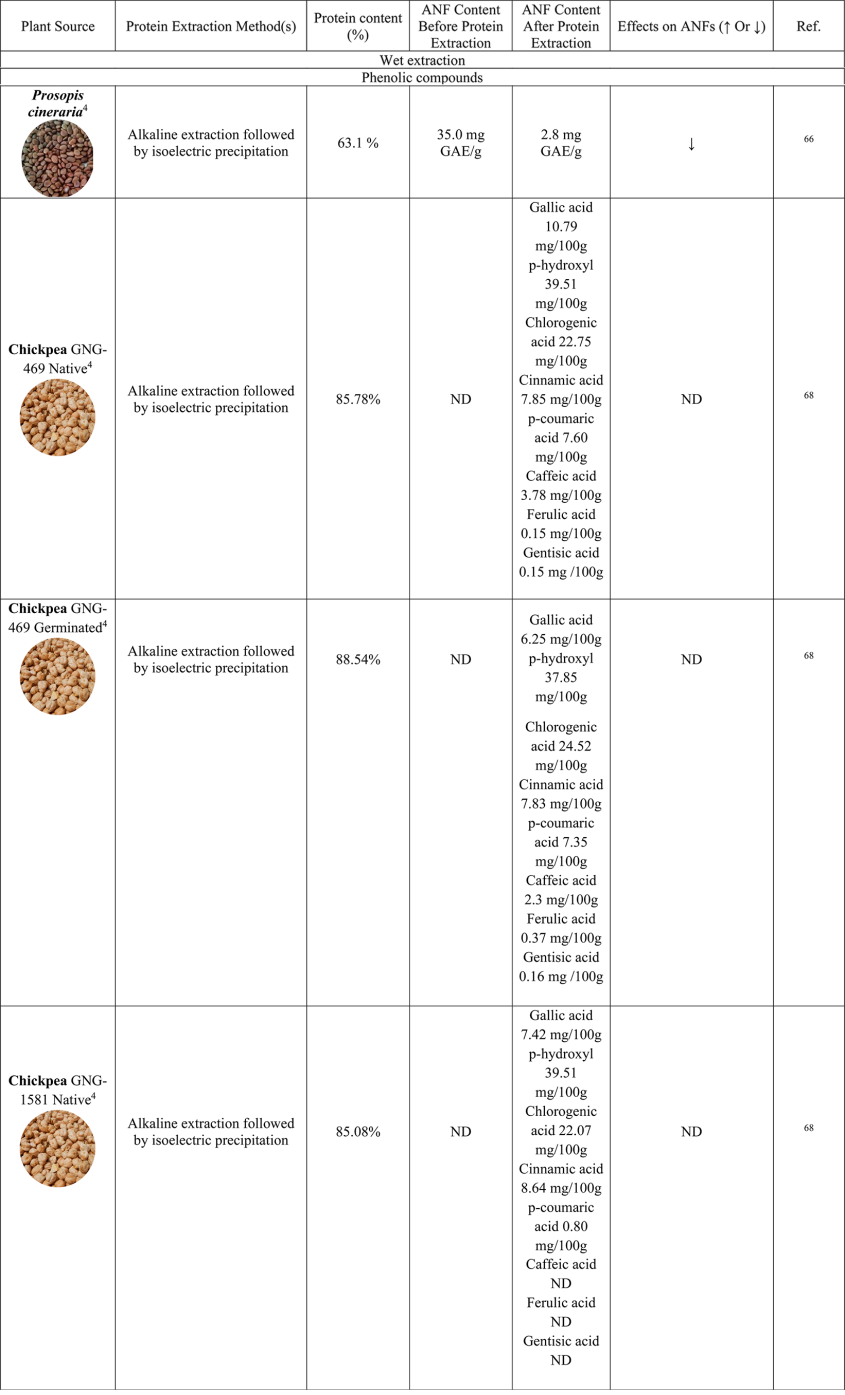

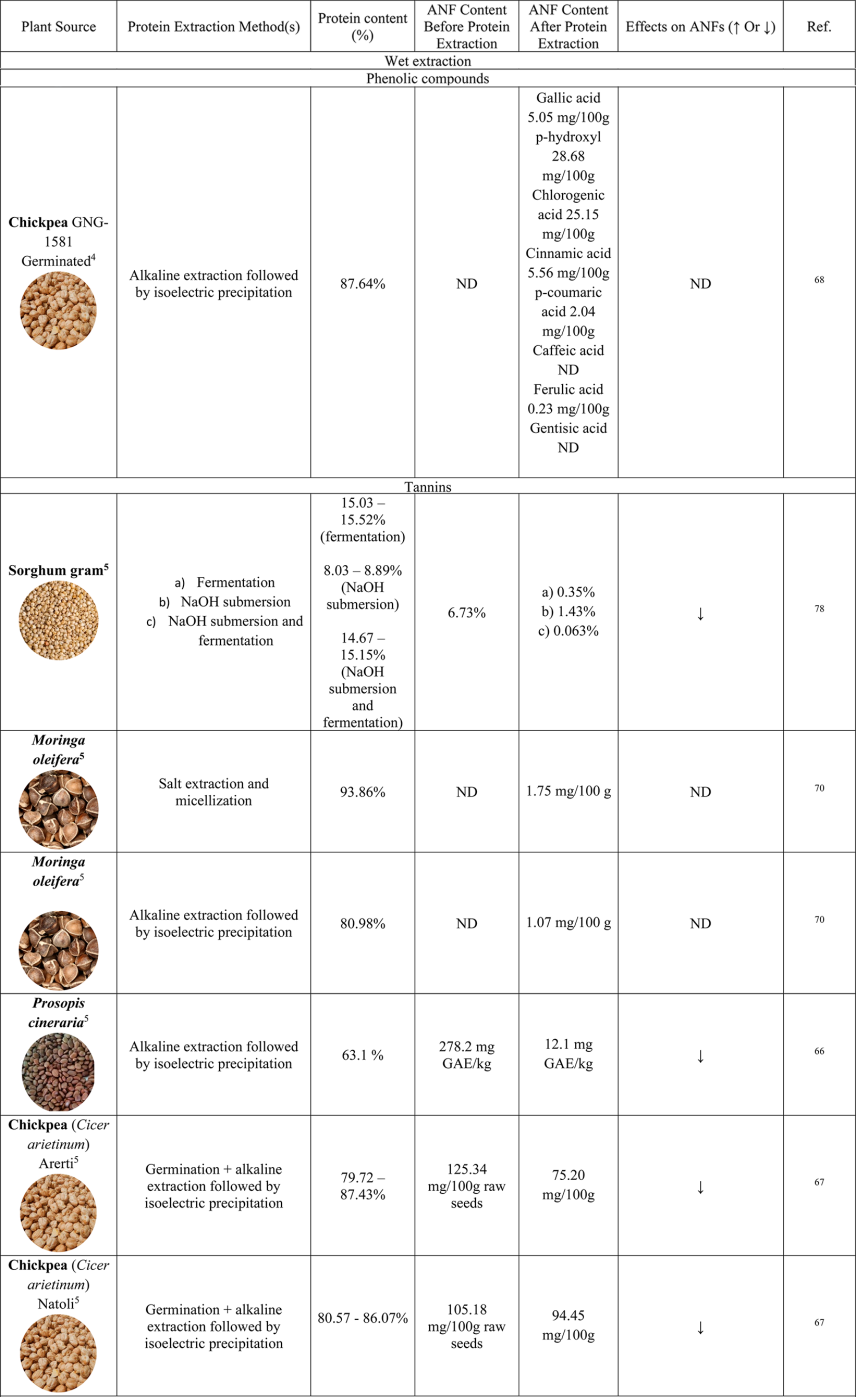

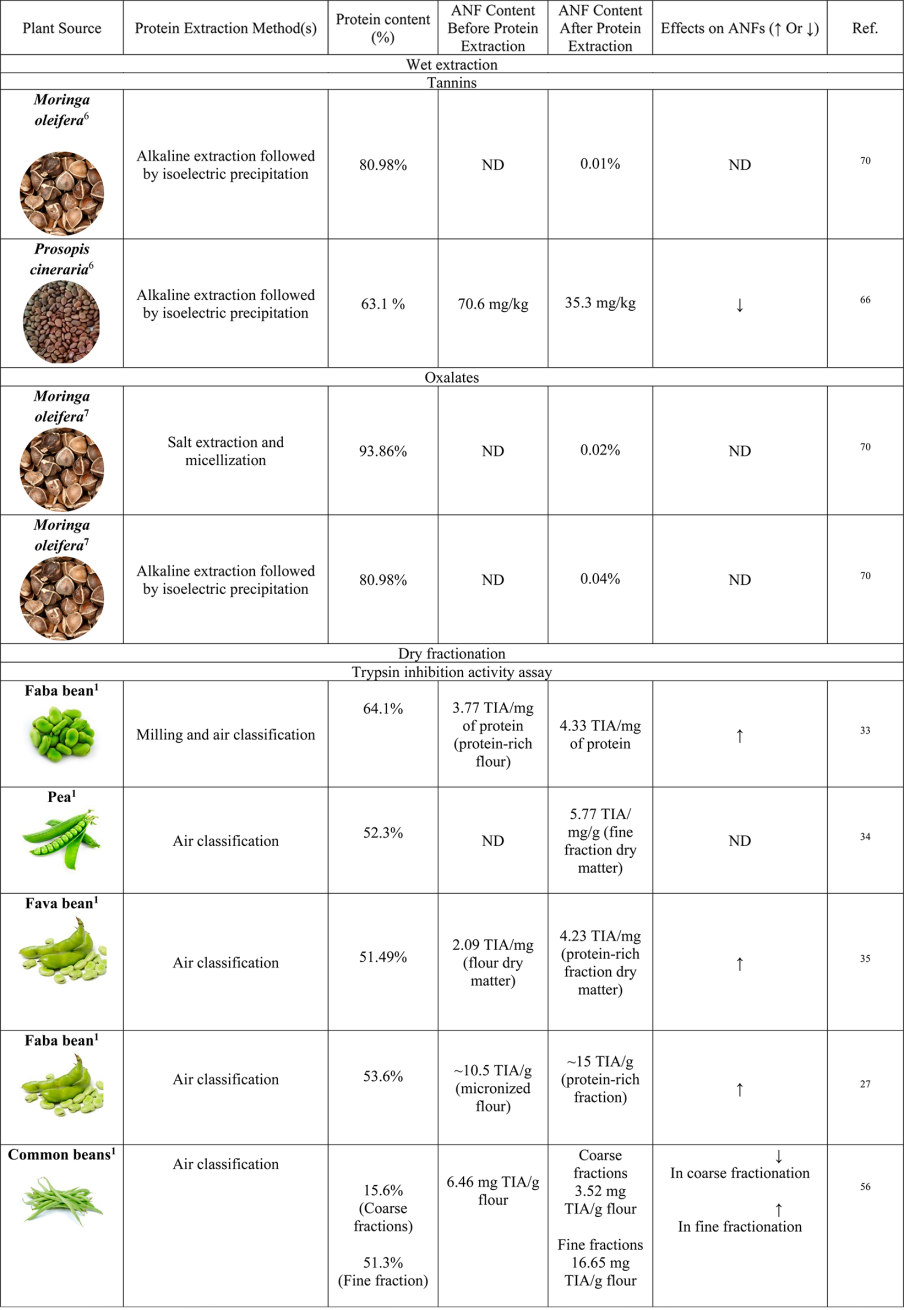

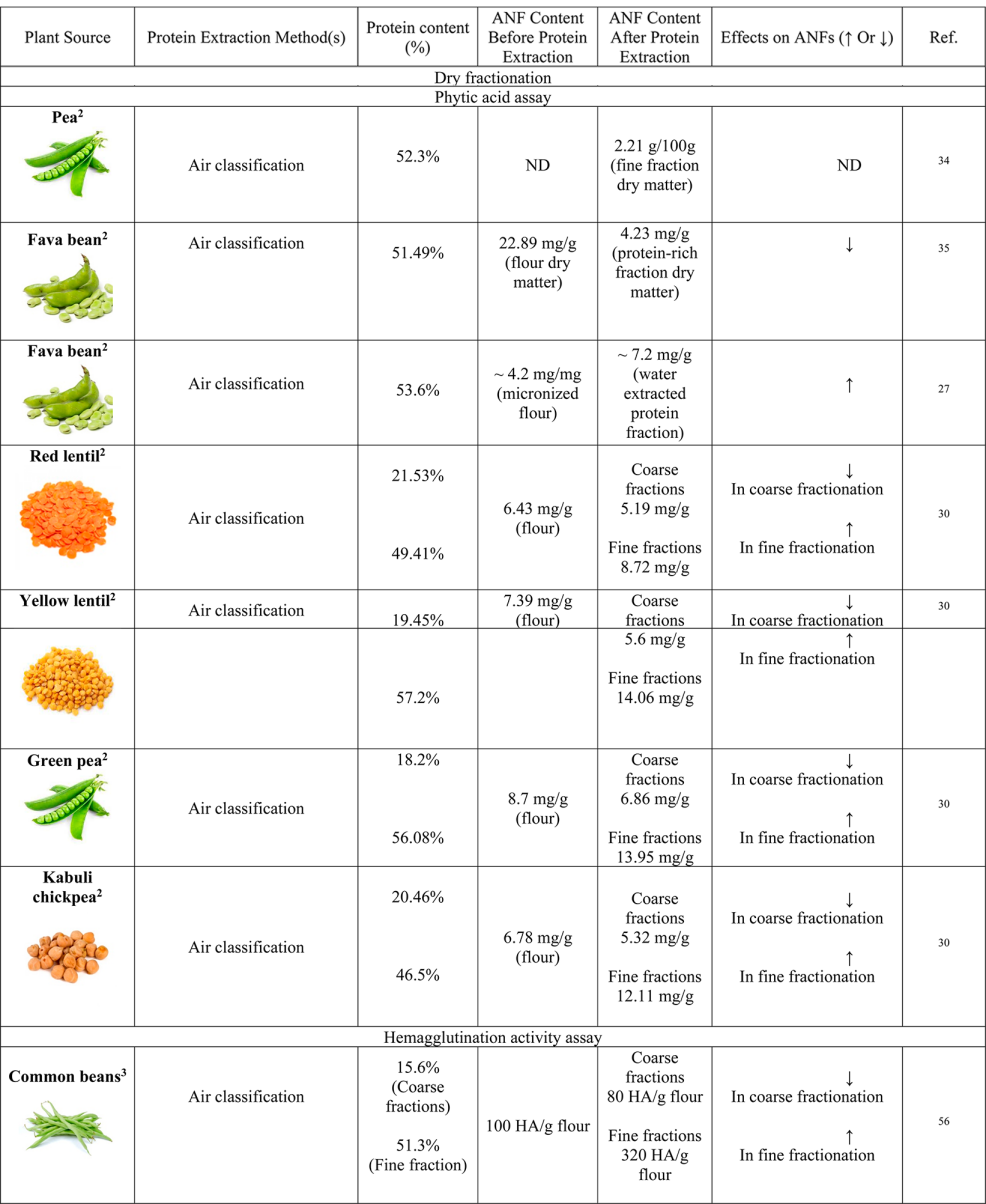
Effect of Wet Protein Extraction and Dry Protein Fractionation on ANFs^1^ Trypsin Inhibition Activity Assay;^2^ Phytic Acid Assay;^3^ Hemagglutination Activity Assay;^4^ Phenolic Compounds;^5^ Tannins;^6^ Saponins;^7^ and Oxalates^a^

a TIA = Trypsin inhibition activity; HU = Hemagglutination unit; HA = Total lectins; GAE = Gallic acid equivalents; and ND = Not detected.

However, regarding the Bowman-Birk trypsin inhibitor, Wang^29^ demonstrated that the soybean Bowman-Birk trypsin inhibitor is composed of two fractions: one fraction eluted at pH 3.5 and another fraction eluted at pH 4.0. Thus, isoelectric precipitation at pH 4.5 can avoid the precipitation of a Bowman-Birk trypsin inhibitor fraction (eluted at pH 3.5), and it is a potential reason for reduced TIA in protein concentrates/isolates. Another aspect that contributes to a lower TIA is the drying method applied after extraction: both freeze-drying and spray drying can lower TIA.^32^

However, the concentration of the trypsin inhibitor differs in plant tissues. Avilés-Gaxiola et al.^31^ discovered that over 90% of TIA in soy and fava beans is concentrated in the cotyledons. Conversely, in chickpeas, TIA is more evenly spread out among various plant parts, with 77.2% to 75.8% in the cotyledons, 11.9% to 15.5% in the embryonic axis, and 10.9% to 8.7% in the seed coat.^33^ This research also highlighted that proteinase inhibitors are found in different cellular locations, including protein bodies, cell walls, intercellular spaces, and the cytosol. Krishnan et al.^32^ noted that in mung beans, TIA is confined to the cytoplasm, avoiding protein bodies.^34^ Thus, given the disparity in concentrations of TIA in different parts of seeds/plants, preprocessing methods such as milling/sieving as well as wet extraction/dry fractionation could have different effects on levels or activity of TIA.

Dry fractionation has been shown to increase trypsin inhibitor (TIA) in various extracted protein ingredients. For example, Vogelsang-O’Dwyer et al.,^33^ Wang and Maximiuk,^34^ Coda et al.,^35^ and Dumoulin et al.^27^ demonstrated that air classification caused the accumulation of the TIA for both fava bean and pea protein rich fraction (Table 2). This can be of interest considering that TIA may provide not only adverse effects on human health but also benefits such as obesity treatment, immunomodulating activities (Bowman-Birk inhibitor), and anti-inflammatory and chemo-preventive properties.^2^ Consequently, depending on the purpose, distinct extraction methodologies may be chosen for tailor-made ingredients. This selection can aim either to mitigate adverse effects through wet extraction or to preserve and amplify potential health benefits via dry extraction.^28,35−37^

### 4.2 Phytic Acid

**Figure 3:**
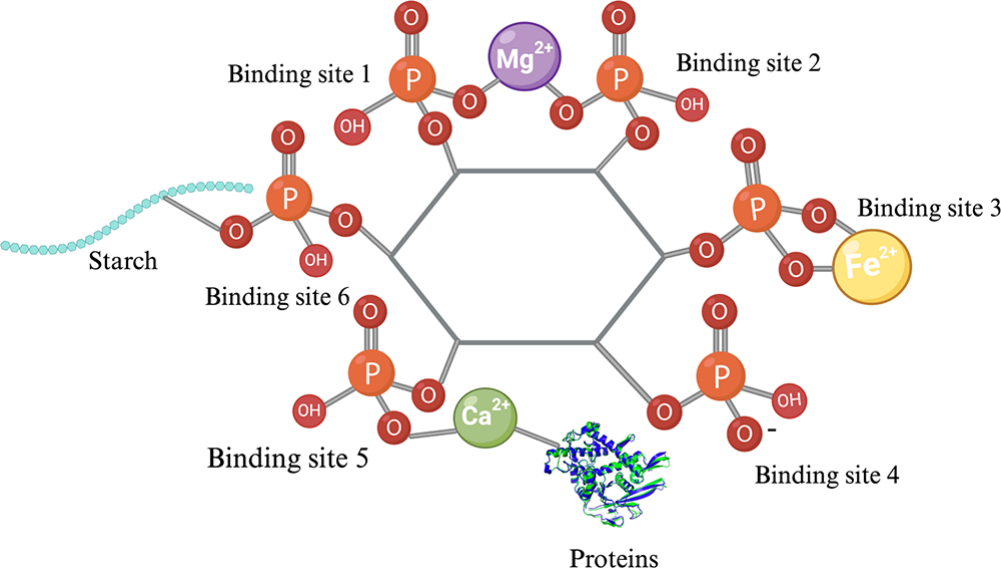
Phytate structure and its’ six binding sites

Phytate (known as Inositol hexakisphosphate) is the salt form of phytic acid. Phytic acid binds with minerals and/or proteins and forms complexes due to the chelating activity of its six reactive phosphate groups (Figure 3).^14^ Traditional methods for reducing phytic acid in plant materials include soaking, cooking, roasting, boiling, germinating, and thermal treatment. Daneluti and Matos^36^ reported that when heated to 150 °C for 1 h, phytic acid is thermally decomposed.^38^ Besides thermal treatment, germination enzymatically hydrolyzes phytic acid, releasing phosphorus, which is used by the plant to grow.^39,38^

Contrary to protein based ANFs such as trypsin inhibitors, protease inhibitors and chymotrypsin inhibitors, phytic acid is less affected by protein extraction. Although Fernández-Quintela et al.^38^ reported that the phytic acid content in protein isolates is lower than in whole seeds (Table 2), the decrease in phytic acid potentially stems from the soaking performed before protein extraction.^40^ Cheng et al.^39^ and Godrich et al.^40^ have demonstrated that soaking can effectively decrease the phytic acid content.^41,42^ Both wet extraction and dry extraction can increase the concentration of phytic acid in protein ingredients. This is due to the unremovable binding between minerals, phytate, and proteins. For example, during isoelectric precipitation, the phytic acid/protein complexes are insoluble and thus recuperated with the precipitated proteins. Although phytic acid cannot be completely removed by alkaline protein extraction, it has been shown that performing ultrafiltration/diafiltration at pH 6 can reduce phytic acid in soy protein isolate when compared to ultrafiltration/diafiltration at pH 9, and isoelectric precipitation at pH 4.5.^43,44^ Similar observations were made by Taherian et al.^43^ in the production of pea protein isolates by membrane processing^45^ and by Mondor et al.^10^ in the production of Desi chickpea protein isolates made from defatted flour. A likely explanation is that the ternary complex (phytic acid, divalent cations, proteins) that can form above the isoelectric point of the proteins (pH 4.5) is weak, allowing significant removal of phytic acid through the ultrafiltration membrane. However, it is hypothesized that protein extraction at extremely acidic conditions can increase the formation of phytate-proteins complexes. At a low pH (pH < 3) binding sites are buried in hydrophobic cores. The denaturization of protein exposes binding sites to phytic acid and minerals.

### 4.3 Lectins (Hemagglutinins)

**Figure 4:**
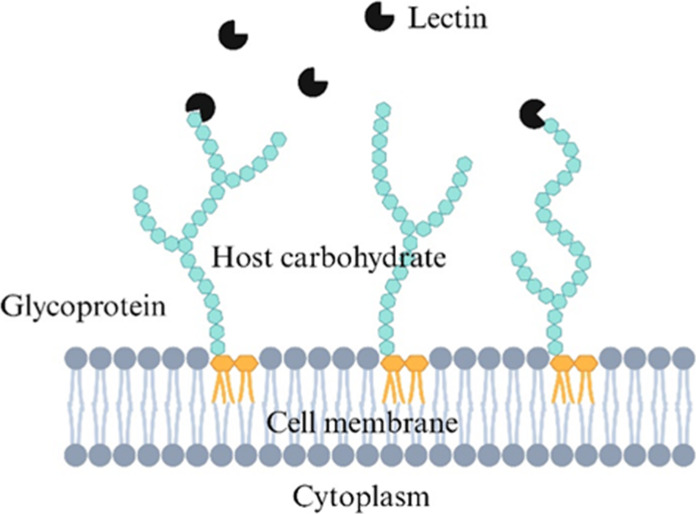
Lectin binding with host carbohydrate complexes from glycoproteins

Lectins, also known as hemagglutinins, are glycoproteins which have been reported in more than 800 legume families as well as in animals (e.g., C-type lectins, galectins, P-type lectins) (Figure 4).^46^ Due to their protein nature, lectins can be denatured/inactivated through high thermal processing. However, it is important to note that the thermal denaturation of lectins is time dependent, and thermal treatment for at least 10 min is needed to inactivate lectins.^47^

Recent research on plant lectins mainly focused on lectins from legumes and wheat germ. Legume lectins tend to show homology in both sequence and structure. The tertiary structural feature of lectins is similar among legumes; they contain a 6- and 7-stranded antiparallel β-sheet. But due to the different types of carbohydrates and different quaternary structure, legume lectins have various carbohydrate specificities and properties.^46,48^ Wheat germ agglutinin shows carbohydrate-binding preference for *N*-acetyl-d-glucosamine and *N*-acetyl-d-neuraminic acid (sialic acid).

Fernández-Quintela et al.^38^ observed that hemagglutination activity was not observable in pea, fava bean, and soybean after protein extraction by alkaline solubilization coupled to isoelectric precipitation.^40^ Although pretreatment steps like soaking and dehulling were taken for pea, fava bean, and soybean seeds, lectins were not removed through the pretreatment steps, as Embaby^47^ demonstrated that both soaking and dehulling cannot decrease hemagglutination activity in lupin seeds. This may be because lectins are located in the inner parts of grains/seeds, thus making them difficult to remove by such processes.^49^ During alkaline solubilization coupled to isoelectric precipitation, Fernández-Quintela et al.^38^ used water as the solubilization medium, and lectins were found to be water-soluble for soybean, pea and *Moringa oleifera* seeds.^50^ So according to Fernández-Quintela et al.,^38^ lectins were not precipitated with the proteins, and so these were found in lower concentration in the protein isolates. Although limited studies have demonstrated that isoelectric precipitation can remove lectins, similar to phytic acid and trypsin inhibitors discussed earlier, lectins can also provide health benefits, such as antimicrobial, antidiabetic, antiproliferative, and antiangiogenic properties.^51^ However, lectin extraction can potentially also be minimized by adjusting the extraction medium, and avoiding choosing a medium such as water or a diluted salt solution (e.g., NaCl, Tris-HCl, PBS), which has an affinity to lectins.^52^

Also, the effects of some novel extraction methods such as microwaves have not been studied in detail. From the limited research available, microwave cooking for 5 to 10 min (depending on different legume seeds) was shown to reduce the hemagglutination activity. For example, Hernandez-Infante et al.^51^ reported that the highest dilution of lectin extract causing agglutination of human erythrocytes from soybean was reduced from 5 to 3 after microwave cooking for 10 min. A similar conclusion was drawn for most of the tested legumes except for chickpea, for which the agglutination of human erythrocytes was not affected by microwave processing.^53^ However, on the basis of the current research, it is difficult to differentiate between the effects of microwave and heat.

Research on protein concentrates and isolates has been limited. Furthermore, there is a noticeable gap in these studies regarding the evaluation of hemagglutination activity against red blood cells, as well as the comparison of immunobinding assays with raw or original flour. For example, Hisayasu et al.^52^ reported the hemagglutination activity in soybean protein isolates (SPI), and heated soybean protein isolates (H-SPI), using hemagglutination activity (red blood cell aggregation assay) and immunobinding assay. The SPI clearly showed hemagglutination activity and immunoreactivity, although the hemagglutination activity was also found in H-SPI, but it was not immunoreactive.^54^

### 4.4 Phenolic Compounds

**Figure 5:**
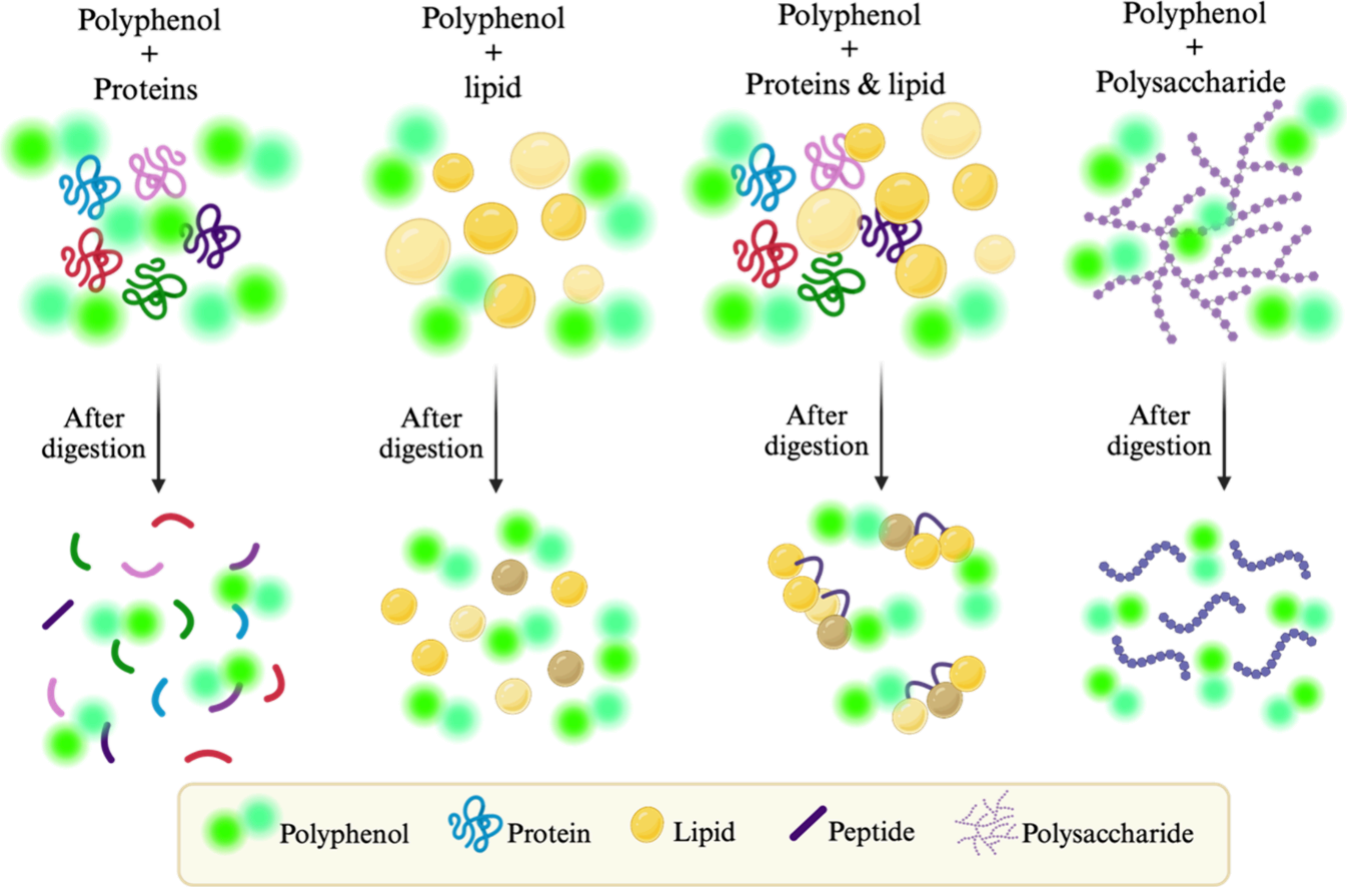
Mechanisms of phenolic compounds with biomolecules after digestion.

**Figure 6:**
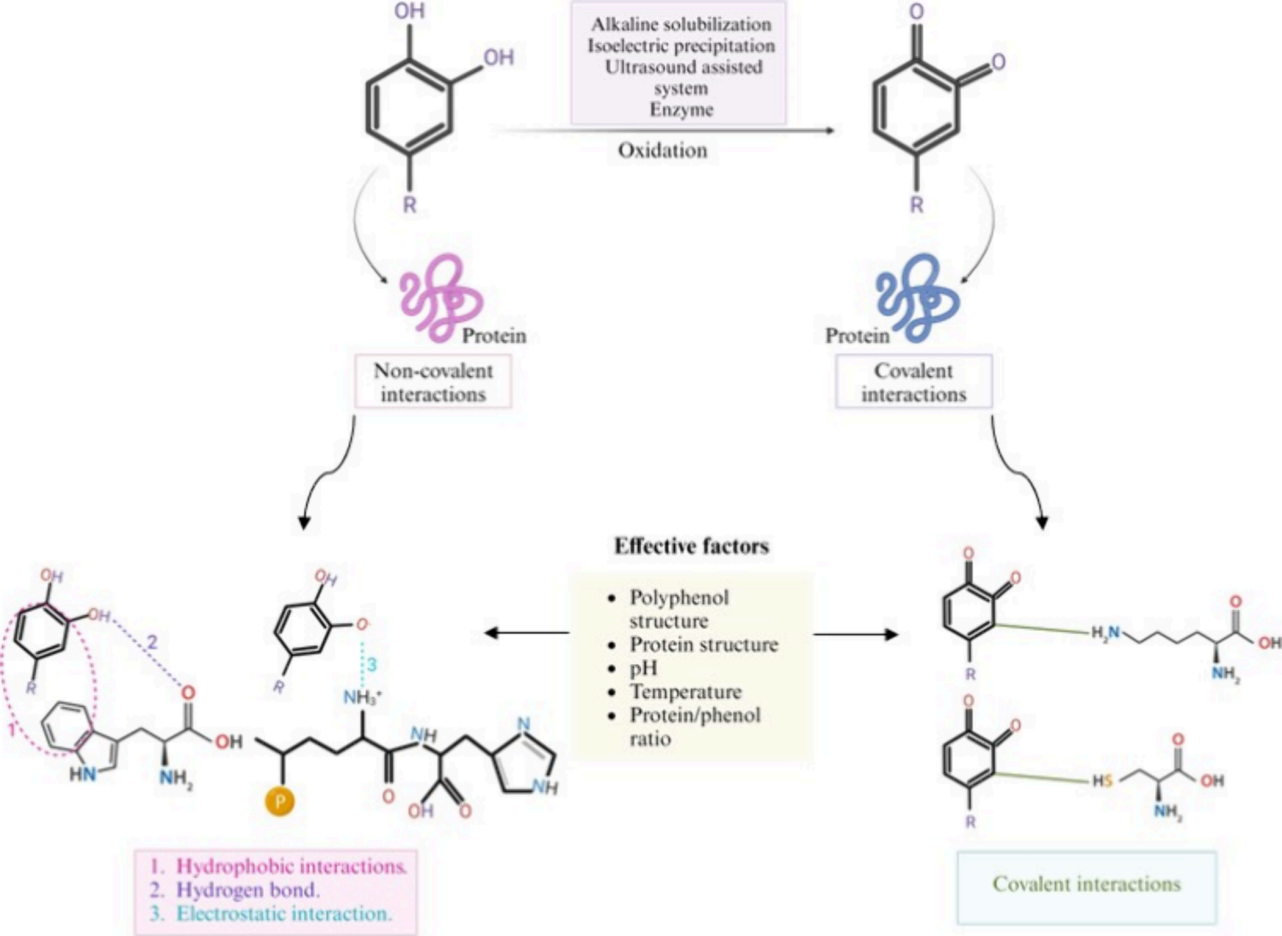
Mechanisms of phenolic compounds with proteins; noncovalent and covalent interactions.

Phenolic compounds are antioxidant compounds that can be divided into three categories: phenolic acids, flavonoids, and tannins. Phenolic compounds possess one or more aromatic rings, coupled to one or more hydroxyl groups.^55^ Thus, phenolic compounds are well-known for their mechanism of action and interactions (Figures 5 and 6), which can be either a hydrogen atom transfer (HAT) mechanism, single electron transfer via proton transfer, or proton loss electron transfer.^56^ As phenolic compounds interact with proteins, this forms a complex that decreases protein digestibility, due to hydrophobic regions that sterically interact and restrict the action of digestive enzymes (Figure 5).^57^ Strauch and Lila,^58^ reported this phenomena on pea protein (pea protein isolate 80%) and cranberry pomace extract, resulting in a decrease of protein digestibility, slower gastric digestion (pepsin <25%) and slower intestinal digestion (pancreatin <35%).^58^

Furthermore, polyphenolic compounds are susceptible to oxidation by molecular oxygen, particularly at alkaline pH, where they interact with the side chain amino groups of peptides, transforming into quinones (Figure 6).^59^ This process facilitates the formation of protein cross-links. The highly reactive nature of these quinones enables them to irreversibly bind with the sulfhydryl and amino groups of proteins.^60^ Additionally, quinones can participate in condensation reactions, culminating in the synthesis of high molecular weight, brown-hued pigments, commonly referred to as tannins. Furthermore, it has been observed that protein-phenolic interactions result in modifications to secondary and tertiary conformations, thermal stability and techno-functional properties.^57^ Concurrently, some studies have found a decrease in protein solubility, while thermal stability may exhibit enhancement.^57^ Furthermore, these interactions potentially lead to a decrease in certain amino acid concentrations and a decrement in protein digestibility.^61^ Additionally, protein-phenolic interactions in protein isolates/concentrates can produce an astringent aftertaste, that can be unpleasant for the consumer.^57,62^ Cosson et al.^63^ reported that 29 phenolic compounds are related with bitterness and or astringency, in pea protein isolates.

Alireza-Sadeghi et al.^64^ reported the reduction of ANFs such as glucosinolates, phytic acid, and phenolic compounds in mustard (*Brassica juncea)* protein isolate (95 g protein/100 g isolate) prepared by isoelectric precipitation, in combination with steam injection heating. After alkaline solubilization of the mustard meal (0.5–2.5 kg in 0.1 mol/L NaCl at a ratio 1:15 (w/v)) at 37 °C for 1 h, the pH was adjusted to 11 and the dispersion was stirred for 30 min at room temperature before the supernatant was recovered by centrifugation. The supernatant pH was adjusted to pH 7.0 with 2 mol/L HCL, activated carbon granules (2% w/v) were added, and then an injection of steam was added to the system to raise the temperature (93 °C), which was followed by cooling and centrifugation. Finally, the precipitate was dispersed in water (1:10 w/v), and the wet protein was neutralized (HCl and/or NaOH), and spray dried, before final protein collection.

Overall, this process reduced ANFs considerably, especially the total phenolic compounds (TPC), which were found to be 1.20 g/100 g in the whole seeds compared to only 0.12g/100g in the protein isolate, yielding a removal rate of >90%. The authors concluded that the loss of TPC is due to dehulling, as mustard hulls are known to contain polyphenols, glucosinolates, and minerals.

Some authors have compared the difference between two or more protein extraction methods, and the impact of these techniques on ANF content. Arntfield et al.^65^ used a micellization technique and high salt protein extraction in fava bean and assessed the behavior of ANFs. Fava bean flour was dispersed (1:10 w/v) in NaCl solution to obtain a protein slurry, and then it was diluted (1:3 w/v) in high salt concentration and decanted. Finally, the protein isolate, in which the protein has a micelle structure, is referred to as protein micellar mass.

The samples obtained by high salt extraction had less protein (56.0 g/100 g), compared to high salt plus micelle extraction (93.8 g/100 g), showing that a process combining both high salt extraction and micelle extraction results in fava bean protein ingredients with higher purity. Nonetheless, TPC in the ingredient resulting from the process combining both high salt extraction and micelle extraction was found to be lower (0.18%), compared to high salt extraction (1.44%). The authors pointed out that the precipitation step during micellization reduced the TPC content (>63%), and only 3.5% of the original TPC remained in the protein isolate. Furthermore, the conditions used in the study were not enough to disrupt hydrogen bonding. As a result, phenolic compounds were not found in the high salt extraction and micellization protein isolate. Moreover, the alkaline pH step could result in some ionization of the phenolic compounds, which will decrease the hydrophobic surface and not favor phenolic interactions, the end result being a protein isolate with reduced TPC.

Mondor et al.^10^ evaluated the composition of two chickpea (Desi and Kabuli) full-fat flours, and protein concentrates prepared by isoelectric precipitation or by ultrafiltration (pH 9)/diafiltration (pH 9 or 6) using a 50 kDa membrane. Protein concentrates prepared by ultrafiltration/diafiltration showed a higher protein content (72.3–82.9 g/100 g), compared to the concentrates prepared by isoelectric precipitation extraction (69.9–78.6 g/100 g). TPC was determined by the Folin-Ciocalteu (FC) method. The results showed that both the ultrafiltration process and the isoelectric precipitation process, in general, significantly decreased the TPC content of the concentrates compared to the process for full-fat flours, with a larger decrease observed for the concentrates prepared by isoelectric precipitation.

Garg et al.^66^ reported a 90% TPC reduction in *Prosopis cinerari* protein concentrate compared to *P. cinerari* seed flour. However, an interesting effect was observed on the antioxidant capacity, which was not affected when compared to *Prosopis cinerari* seed flour, which had 3.2 mg AAE/g, while the protein concentrate had 3.0 mg AAE/g. The authors pointed out that *P. cinerari* seeds are rich in phenolic compounds, while the protein concentrate has lower TPC, and still showed fair antiradical scavenging capacity. During alkaline extraction, the observed interconversion between sulfhydryl and disulfide bonds may elucidate the cause of the increased phenolic content (antioxidant capacity, AOX) as reported by Garg et al.^66^

Mesfin et al.^67^ studied two chickpea varieties (Natoli and Arerti) and applied two pretreatments prior to protein extraction. In the first pretreatment, chickpea seeds were roasted at two temperatures (150 °C and 180 °C), and proteins were extracted by alkaline solubilization and isoelectric precipitation. In the second pretreatment, the seeds were germinated for different durations (24, 48, and 72 h) and then proteins were extracted by isoelectric precipitation. For both varieties and treatments, phenolic compound increased by >86.0% for Arerti, and >62.2% for Natoli. Additionally, the initial TPC in the Arerti variety was 179.8 mg GAE/100 g. The highest TPC observed in the protein extracted/roasted at 180 °C pretreatment was 311.8 mg GAE/100 g, while for the Natoli variety, the initial TPC in the seeds was 186.0 mg GAE/100 g. The highest TPC observed in the protein extracted/germinated for 72 h pretreatment was 328.5 mg GAE/100 g. These results were correlated with antioxidant activity and showed that TPC has a correlation with DPPH inhibition; consequently, this trend was not observed in a hydrogen peroxide scavenging assay. Roasting can affect TPC, as this technique favors the formation of compounds due to Maillard reactions, which could explain why the TPC is higher for seeds roasted at 180 °C than at 150 °C. Finally, TPC increased with the germination duration, and the solubilization of some compounds such as tannins could be the possible reason for the increase observed during the germination process. These findings are similar to those reported by Sofi et al.,^68^ who studied four varieties of chickpea: GNC 469 Native, GNC 469 Germ, GNC 1581 Native, and GNC 1581 Germ. In this study, the protein extraction was performed with alkaline solubilization and isoelectric precipitation. Additionally, the identification of phenolic compounds was assessed by HPLC, finding higher concentrations of chlorogenic acid (24.52–25.15 mg/100 g), ferulic acid (0.37–0.23 mg/100 g), and gentisic acid (0.16 mg/100 g) in a sample of germinated chickpea cultivars (GNC 469 and GNC 1581) compared to the other varieties of chickpea. The authors concluded that increased levels of TPC could be due to the liberation of some enzymes such as amylolytic, proteolytic, and lipolytic enzymes that are produced during germination. However, phenoloxidase and peroxidase enzymes could be responsible for catalyze the oxidation of some phenolic substrates, leading to a decrease in some phenolic acids.

Wang et al.^61^ reported the effect on TPC, after protein extraction by isoelectric precipitation, for two varieties of *Salvia hispanica* (chia) from Mexico and Great Britain. The resulting protein concentrates obtained from the Mexican and British varieties had 88.32 g/100 g and 89.20 g/100 g of protein, respectively. Furthermore, Wang et al.^61^ isolated the major protein fractions from *S. hispanica*; albumins extracted from the Mexican and British varieties showed the highest TPC with 4884 mg GAE/100 g and 3338 mg GAE/100 g, respectively. These values are higher than the ones observed for the protein concentrates at 248.34 mg GAE/100 g and 306.99 mg GAE/100 g for the British and Mexican varieties, respectively. However, globulin fraction from both varieties showed a low TPC with 209.94 mg GAE/100 g and 213.55 mg GAE/100 g for the Mexican and British varieties, respectively. Wang et al.^61^ concluded that processing conditions such as particle size, pressure, temperature, and different solvent selection will affect the concentration and solubility of TPC. Meanwhile, albumin fraction had higher TPC, as albumins are water-soluble proteins, compared with globulins from chia. Furthermore, protein-phenolic interactions can have a negative impact over techno-functionality, protein quality and digestibility. Additionally, this study showed that *in vitro* protein digestibility (IVPD) in both protein concentrates was not affected by ANFs.

### 4.5 Oxalates

**Figure 7:**
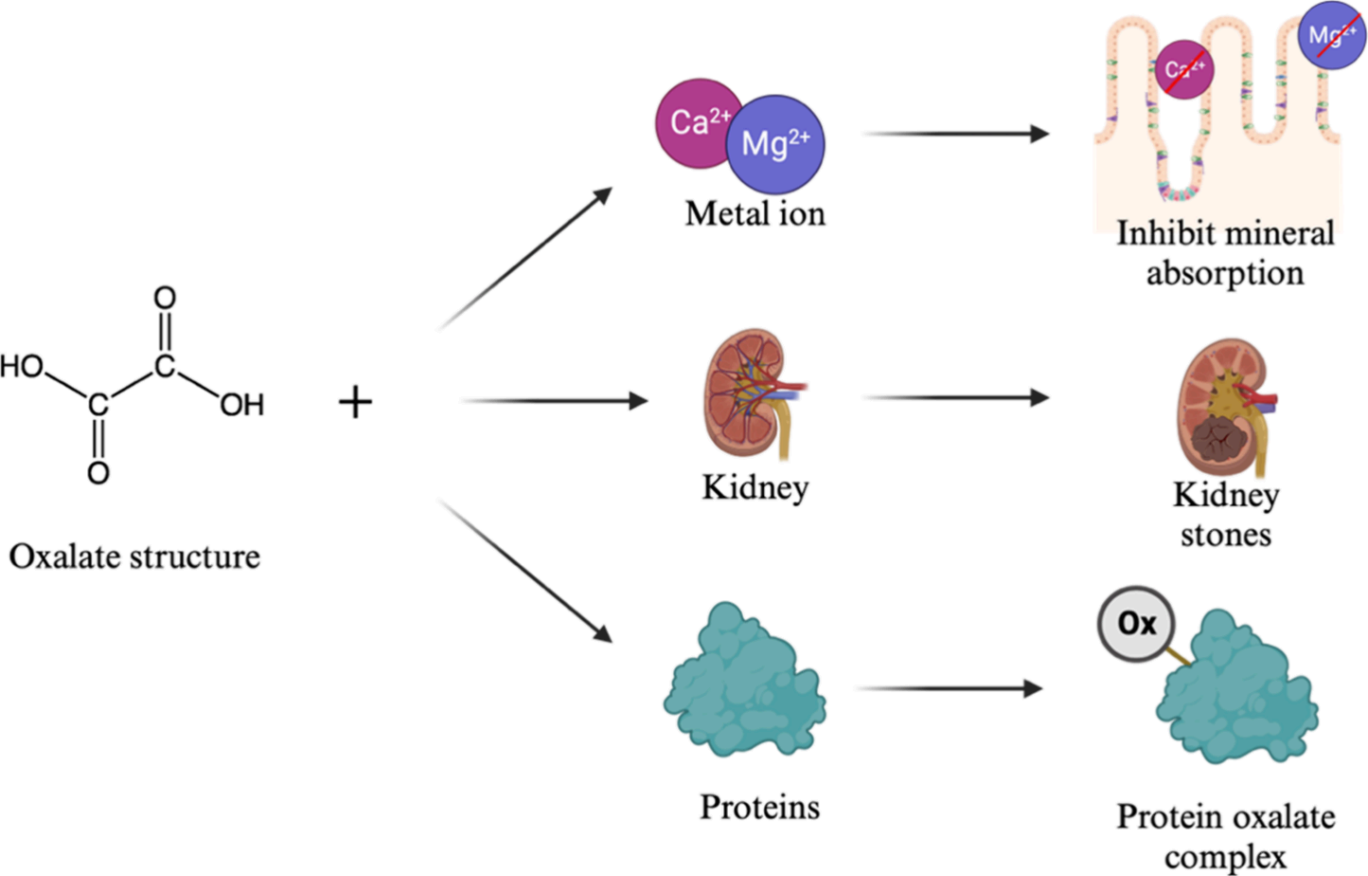
Mechanisms of oxalates binding to different compounds

Oxalates, also known as oxalic acid, are organic compounds that can form water-soluble salts (Na^+^, K^+^, and NH) and water-insoluble salts (Ca^2+^, Fe^2+^, and Zn^2+^) (Figure 7). Thus, when oxalates bind with minerals in the small intestine, these nutrients are unable to be absorbed. Because of this, oxalates are mostly toxic, and are the main cause of kidney stones.^69^ llingworth et al.^70^ reported an oxalate content of *Moringa oleifera* protein isolates for two extraction processes, alkaline extraction/isoelectric precipitation, and salt extraction/micellization, with respective values of 0.04% and 0.02%.

Furthermore, it is important to highlight that there is a lack of current research regarding the health benefits of oxalates, particularly in relation to their presence in protein ingredients produced from soybeans, walnuts, potatoes, cereals, and green leafy vegetables.

### 4.6 Saponins

**Figure 8:**
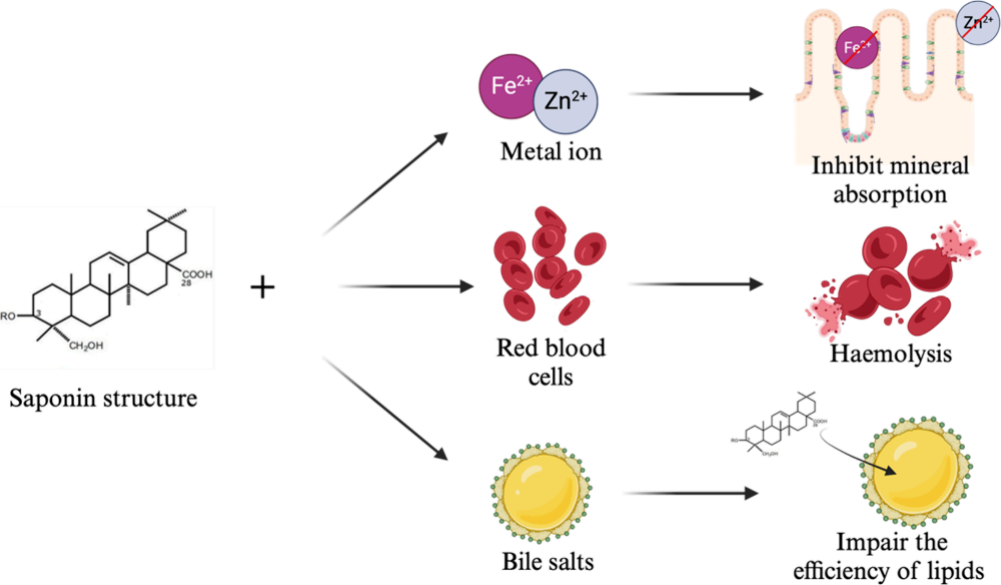
Mechanisms of saponin binding to different compounds

Saponins are steroidal or triterpene glycosides. These compounds are mostly soluble in water and ethanol solutions.^55,71^ The mechanism of action of saponins is shown in Figure 8. First, saponins can form complexes with minerals such as Fe^2+^ and Zn^2+^ and reduce their bioavailability in the intestinal tract. Second, saponins produce hemolysis in red blood cells in the human body. Finally, when saponins bind with bile salts, these inhibit the functionality of lipid function.^72^ However, saponins could be used to reduce cardiovascular diseases and prevent heart attacks because of their hemolytic activity.^14,69^ Additionally, saponins can lower plasma cholesterol (LDL).^73^ In the food industry, saponins are used for their foaming capacity and emulsifying properties.

Garg et al.^66^ studied the difference in ANFs in *Prosopis cineraria* seed flour and protein concentrate. They observed a 50% reduction in saponin content when proteins were solubilized at different pH (8 to 10), at different times (1 to 3 h) and temperatures (30 to 60 °C), and finally recovered by isoelectric precipitation, compared to the flour. The authors inferred that the extraction process decreased the saponin content, and that the initial concentration of saponins in *P. cineraria* seeds was low. The observed reduction in saponin content during protein extraction could be explained by the fact that saponins are polar compounds that are soluble in polar solvents such as water.^14^

Illingworth et al.^70^ reported no effect over saponin content in *Moringa oleifera* protein isolates, using two different extraction methods. First, alkaline extraction/isoelectric precipitation was assessed. Flour was dispersed in deionized water (1:10 w/v) and adjusted to different pH (7.5, 8.5, 9.5, 10.5 and 11.5), extracted for different times (10, 20, 30, 40, 50, 60 min) and temperatures (30, 40, 50, 60, 70 °C), and the most effective parameters were selected (pH 8.5 for 10 min at 40 °C). Second, salt extraction and micellization was used to extract proteins from *M. oleifera* flour with different extraction parameters. The authors selected 0.5 mol L^–1^ NaCl for 10 min at 40 °C as optimal conditions, with a ratio of 1:10 (w/v) of sample. Saponin content in the isolates was found to be 0.1% for both methods.

### 4.7 Tannins

**Figure 9:**
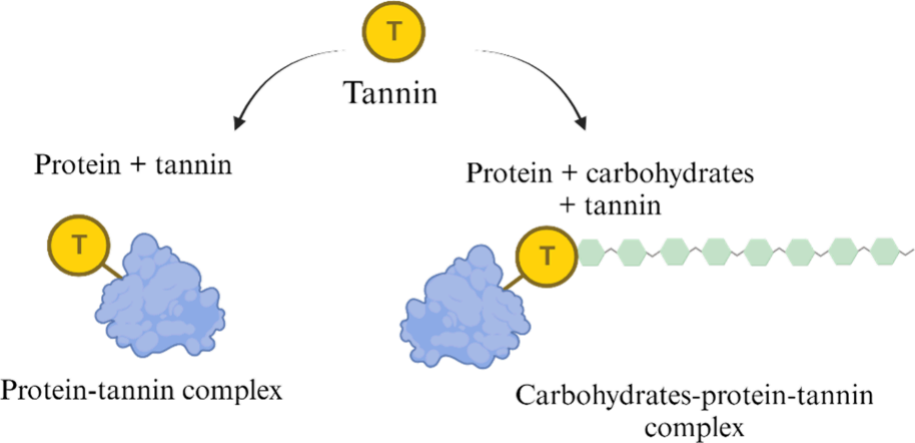
Mechanisms of tannins binding to proteins and carbohydrates

Tannins are water-soluble compounds and can be classified as hydrolyzable, condensed, and complex tannins.^74,75^ They belong to the phenolic compounds family. Tannins can form complexes with proteins through hydrogen bonds, hydroxyl, and carbonyl groups. In addition, they can precipitate proteins in aqueous solutions (Figure 9),^76^ and they inhibit amylase activity and iron absorption and storage.^77^ Despite this, tannins possess similar positive properties such as anti-inflammatory, anticancer, antiviral, and antimicrobial.^74^

Garg et al.^66^ reported that tannins can be reduced by 95% in protein concentrate from *Prosopis cinerari* seeds, by using isoelectric precipitation. The authors concluded that tannin content was less than in other legumes and inferred that the extraction system employed reduced ANFs, and additionally dehulling and defatting by cold extraction could help to reduce certain ANFs such as tannins and phytic acid. Mesfin et al.^67^ compared Natoli and Arerti chickpea varieties, in terms of the tannin content in their corresponding protein concentrates, produced by germination and isoelectric precipitation. The results showed that tannin content decreased in both concentrates from 125.34 mg/100 g in the raw sample to 41.90 mg/100 g (germinated 72 h) and 62.74 mg/100 g (roasted 180 °C) for the Natoli variety. Meanwhile, Arerti went from 105.18 mg/100 g in the raw sample to 41.83 mg/100g (germinated 72 h) and 63.0 mg/100 g (roasted 180 °C). These decreases in tannin content may result from leaching from the chickpea into the soaking water, or from hydrophobic association of tannins with the seed proteins and enzymes.

Illingworth et al.^70^ compared the effect of alkaline extraction/isoelectric precipitation, and salt extraction/micellization on *Moringa oleifera* protein isolates. The parameters selected for alkaline extraction and isoelectric precipitation were pH 8.5 for 10 min at 40 °C. Then, for salt extraction and micellization, the parameters were 0.5 mol L^–1^ NaCl for 10 min at 40 °C, which resulted in the highest protein extractability. Tannin content decreased from 1.75 mg/100 g in micelle protein isolation to 1.07 mg/100 g in alkaline solubilization/isoelectric precipitation; as a result no statistically significant difference was observed (*p* > 0.05). Gunawan et al.^78^ reported the effect of sorghum fermentation with different bacteria (*L. bulgaricuss, L. casei*, and *L. brevis*) on tannin and protein content. The tannin content decreased in all processes. Initial tannin content in raw sorghum was 6.73% and 6.16% in peeled sorghum. Tannin content was measured using the fermentation process (0.36%), NaOH submersion process (1.43%), and NaOH submersion followed by fermentation (0.063%). The authors concluded that the fermentation process significantly decreases tannin concentration; nonetheless, the content is still higher than 0.3%, which is the standard value suggested by the FAO/WHO.^79,80^ Of note, the third process (NaOH submersion followed by fermentation) showed the lowest amount of ANFs, which is attributed to soaking of the seeds with NaOH. Furthermore, the dehulling process can reduce tannins, as these are present in the external coat of seeds.

### 4.8 Other ANFs

There are other ANFs that have been overlooked, or there is scarce information on anthocyanins, flavonoids, cyanogenic glycosides and glucosinolates. Alireza et al.^64^ reported on glucosinolate content in protein isolates prepared by alkaline extraction/isoelectric precipitation and steam injection of *Brassica juncea* (mustard) seeds. Isothiocyanate content was reduced from 10.20 mg/g in the whole seed to 0.44 mg/g in the protein isolate, while 5-vinyloxazolidine-2-thione content was measured at 7.85 mg/g in the whole seed and was not detected in the protein isolate. During gastrointestinal digestion, digestive enzymatic hydrolysis of glucosinolates produces undesirable and toxic components such as isothiocyanates and oxazolidine thione. Alireza et al.^64^ noted that the reduction of these components in *B. juncea* protein isolate might be related to the addition of activated carbon and thermal coagulation in the protein extraction steps, and the washing during isolation of protein. However, Traka^81^ reviewed the effects of glucosinolates and found epidemiological evidence that implies that eating foods rich in glucosinolates is associated with a lower risk of having a myocardial infarction and different types of cancer (lung, stomach, breast, colorectal, bladder, and prostate).^81^

Mesfin et al.^67^ studied protein isolates from two chickpea varieties (Arerti and Natoli) treated at different roasting temperatures (150 °C and 180 °C) and germination times (24, 48, and 72 h). The results showed that flavonoid content increased in both varieties for all the treatments. The Arerti variety went from 68.2 mg CEQ/100 g in raw seed to 185.4 mg CEQ/100 g in germinated seeds (72 h), while the Natoli variety increased from 88.3 mg CEQ/100 g in raw seed to 197.6 mg CEQ/100 g in germinated seeds (72 h). The germination process produced the highest flavonoid content in both chickpea varieties. The authors attributed this phenomenon to enzymatic biosynthesis of flavonoids from the germination of seed coats and cotyledons. Flavonoids are the major antioxidant agent of the phenolic family. They possess the ability to prevent ROS (reactive oxygen species) formation. This highly scavenging activity stems from their hydroxyl groups or substituents. Furthermore, it has been reported that flavonoids have cardioprotective, chemo-preventive, antimicrobial, and antidiabetic effects. Similarly, anthocyanins have shown antioxidant activity, and they can even modulate glucose metabolism.^82^

Arntfield et al.^65^ reported the content of two pyrimidine glycosides, vicine and convicine, in *Vicia faba* protein isolates from high salt extraction, and high salt extraction followed by micellization. After high salt extraction, vicine content increased from 12.20 mg/g to 13.45 mg/g, while convicine content increased from 5.70 mg/g to 6.29 mg/g. Despite this, when high salt extraction was combined with micellization, the content of each compound decreased by 0.64 mg/g for vicine and 0.28 mg/g for convicine. These compounds are similar to other ANFs, as they are highly soluble in extraction media. Thus, alkaline solubilization, isoelectric precipitation and micellization are effective methods for removing these compounds. Pyrimidine glycosides reduce glutathione and glucose-6-phosphate dehydrogenase activity, which can result in hemolytic anemia.^83^ Additionally, it is important to highlight that there is a lack of current research regarding the health benefits of pyrimidine glycosides, and all studies are focused on the negative effects and the risk of favism.^84^

## 5 Strategic Foresight

ANFs typically exhibit both detrimental and beneficial effects on human health. For instance, certain protein extraction methods, such as dry extraction, are unable to eliminate specific ANFs like trypsin inhibitors. Additionally, phytic acid forms stable complexes with proteins that are difficult to remove by both wet and dry extraction methods even if ultrafiltration/diafiltration at pH 6 has shown some promising results. In the case of lectins, the studies demonstrated that isoelectric precipitation at a certain pH can prevent lectins from being precipitated in the final products. Furthermore, in most cases, wet extraction methods such as alkaline solubilization/isoelectric precipitation have been shown to decrease the content of phenolic compounds. Regarding tannins, as these are water-soluble compounds, they can be removed using protein extraction methods. Additionally, dehulling and soaking seeds as a pretreatment can be effective in reducing these ANFs. For saponins, significant reduction can be achieved through alkaline solubilization and salt extraction; soaking the seeds or grains prior to extraction can increase saponin removal, as saponins leach out into soaking liquor. Studies on oxalates have been relatively limited; however, it can be inferred that salt extraction and isoelectric precipitation might be effective in reducing the content of these compounds. Concerning novel protein technologies, there is a lack of studies demonstrating their effect on ANFs such as cyanogenic glycosides, glucosinolates, anthocyanins, and goitrogens. Consequently, the presence of ANFs in plant proteins may confer potential health benefits. Therefore, investigating whether protein extraction methods can enhance these benefits is a worthwhile area of study.

## Abbreviations Used

ANFs: Antinutritional factors
AOX: Antioxidant capacity
BPAs: Bisphenol A
CEQ: Catechin equivalents
FC: Folin-Ciocalteu
GAE: Gallic acid equivalents
HA: Total lectins
HAT: Hydrogen atom transfer
HU: Hemagglutination unit
IVPD: In vitro protein digestibility
LDL: Lower plasma cholesterol
ROS: Reactive oxygen species
TI: Trypsin inhibitor
TIA: Trypsin inhibition unit
TPC: Total phenolic compounds
